# Knee Moment-Angle Characteristics and Semitendinosus Muscle Morphology in Children with Spastic Paresis Selected for Medial Hamstring Lengthening

**DOI:** 10.1371/journal.pone.0166401

**Published:** 2016-11-18

**Authors:** Helga Haberfehlner, Richard T. Jaspers, Erich Rutz, Jules G. Becher, Jaap Harlaar, Johannes A. van der Sluijs, Melinda M. Witbreuk, Jacqueline Romkes, Marie Freslier, Reinald Brunner, Huub Maas, Annemieke I. Buizer

**Affiliations:** 1 Laboratory for Myology, Department of Human Movement Sciences, Faculty of Behavioural and Movement Sciences, Vrije Universiteit, Amsterdam, The Netherlands; 2 Department of Rehabilitation Medicine, VU University Medical Center, Amsterdam, The Netherlands; 3 MOVE Research Institute Amsterdam, The Netherlands; 4 Pediatric Orthopaedic Department, University Children’s Hospital Basle (UKBB), Basle, Switzerland; 5 Laboratory for Movement Analysis, University Children's Hospital Basle (UKBB), Basle, Switzerland; 6 Department of Orthopaedic Surgery, VU University Medical Center, Amsterdam, The Netherlands; IRCCS E. Medea, ITALY

## Abstract

To increase knee range of motion and improve gait in children with spastic paresis (SP), the semitendinosus muscle (ST) amongst other hamstring muscles is frequently lengthened by surgery, but with variable success. Little is known about how the pre-surgical mechanical and morphological characteristics of ST muscle differ between children with SP and typically developing children (TD). The aims of this study were to assess (1) how knee moment-angle characteristics and ST morphology in children with SP selected for medial hamstring lengthening differ from TD children, as well as (2) how knee moment-angle characteristics and ST morphology are related. In nine SP and nine TD children, passive knee moment-angle characteristics and morphology of ST (i.e. fascicle length, muscle belly length, tendon length, physiological cross-sectional area, and volume) were assessed by hand-held dynamometry and freehand 3D ultrasound, respectively. At net knee flexion moments above 0.5 Nm, more flexed knee angles were found for SP compared to TD children. The measured knee angle range between 0 and 4 Nm was 30% smaller in children with SP. Muscle volume, physiological cross-sectional area, and fascicle length normalized to femur length were smaller in SP compared to TD children (62%, 48%, and 18%, respectively). Sixty percent of the variation in knee angles at 4 Nm net knee moment was explained by ST fascicle length. Altered knee moment-angle characteristics indicate an increased ST stiffness in SP children. Morphological observations indicate that in SP children planned for medial hamstring lengthening, the longitudinal and cross-sectional growth of ST muscle fibers is reduced. The reduced fascicle length can partly explain the increased ST stiffness and, hence, a more flexed knee joint in these SP children.

## Introduction

Children with spastic paresis (SP) who are walking with a flexed knee gait pattern are frequently treated by single-event multilevel surgery (SEMLS) [1, 2]. In such a surgery, several bony and soft-tissue procedures are combined in a single session. One commonly used surgical soft-tissue procedure is medial hamstring lengthening [3–10]. In a high number of children with SP, SEMLS including medial hamstring lengthening, seems successful in correcting the flexed knee gait pattern [8, 11–13]. Side effects on gait, however, due to weakening of the hamstring muscles and overcorrections leading to hyperextension of the knee, increased anterior pelvic tilt, and lumbar lordosis, are frequently reported [6, 8, 10, 14, 15]. Also, persistence of flexed knee gait or even recurrence after initial success can be a problem [6, 8, 15]. Several strategies have been proposed to improve the selection safety of patients for surgical medial hamstring lengthening to reduce side effects. Botulinum toxin test injections have been recommended to assess possible negative effects of muscle weakening on gait [16]. Also, musculoskeletal modeling, used to estimate muscle length changes during gait, has been proposed to assist decision making by orthopedic surgeons [17–19]. However, with musculoskeletal modeling generally only origin-insertion length of a muscle (i.e. length of the muscle-tendon unit (MTU)) is estimated based on joint angles and moment arms. This does not provide full insight in morphological alterations that may potentially underlie the reduced range of motion (ROM) around the knee in children with SP. Assessment of tendon length, fascicle length, and physiological cross-sectional area (PCSA) by ultrasound allows to relate alterations in muscle morphology to limitations in knee ROM and to increased joint stiffness (e.g. a smaller PCSA will decrease while shorter fascicles will increase passive MTU stiffness). Such information may provide indications for the magnitude of effects to be achieved by hamstring lengthening. To our knowledge, such measurements of fascicle length, PCSA and tendon length of hamstring muscles have not been performed in children with SP indicated for medial hamstring lengthening. Insight in morphological variables that affect knee-joint mechanics in children with SP prior to such surgery is the first step to identify factors that explain the side effects.

One of the targeted muscles for medial hamstring lengthening is the biarticular semitendinosus muscle (ST). The ST is divided by a tendinous inscription into two in series arranged compartments which are separately innervated [20–25]. Because of its low degree of pennation, the ST exerts force over a large ROM in hip and knee joints [25]. Low muscle belly volume and short muscle belly length of ST have previously been shown for SP children [26–29]. We have recently described a freehand three-dimensional ultrasound method (3D US) to assess morphological variables of ST (e.g. muscle belly length, tendon length, fascicle length and whole muscle volume, and volumes and fascicle length of both compartments) [30] and a method to reliably measure knee moment-angle characteristics in children with SP [31], which provides quantitative measures of knee ROM and stiffness.

The aims of this study were to assess how (1) knee moment-angle characteristics and ST morphology in children with SP selected for medial hamstring lengthening differ from those in age-gender matched typically developing (TD) children, as well as (2) how knee moment-angle characteristics and ST morphology are related.

We hypothesized that in children with SP knee moment-angle curves are steeper and shifted to more flexed knee angles in comparison to TD children. Furthermore, we expected that these differences are explained by differences in morphological properties of ST.

## Methods

The study was approved by the Medical Ethics Committees of the VU University Medical Center (VUmc), Amsterdam (The Netherlands) and of the University of Basel Children’s Hospital (UKBB), Basel (Switzerland). All children and their parents gave written informed consent.

### Study population

We recruited children with SP at the pediatric rehabilitation and orthopedic departments of the VUmc and the pediatric orthopedic department of the UKBB who were selected for SEMLS to improve gait. Six children with SP were recruited and assessed at the VUmc and three children with SP at the UKBB. All TD children were recruited and assessed at the VUmc. Included patients had: (1) a clinical diagnosis of SP due to cerebral palsy or hereditary spastic paresis [32, 33], (2) were selected for ST lengthening within a SEMLS or as a single procedure—indications for surgery were (a) a fixed knee flexion contracture of ≥15° and/or a popliteal angle of ≥60° and (b) a gait pattern with flexion of the knee in midstance and endorotation-adduction movement of the hips in terminal swing, (3) Gross Motor Function Classification System (GMFCS) [34] level I, II (walking without walking aids) or III (walking with a walking aid), and (4) were between 6 and 20 years old. Patients were excluded if they had interfering treatment and/or had a co-morbidity that could affect walking ability and tissue properties of the hamstring muscles. We considered as interfering treatment: (1) medication that affected neuromuscular properties, (2) treatment with Botulinum toxin A, or (3) serial casting within three months prior to the measurements, (4) selective dorsal rhizotomy, (5) any preceding hamstring muscle surgery, or (6) intrathecal baclofen treatment. The control group consisted of age and gender matched TD children. Table 1 shows the subject characteristics. Each group consisted of 5 females and 4 males with a mean age of 14 years/1 month. Seven children with cerebral palsy and two children with hereditary spastic paresis were included in the patient group. Children with hereditary spastic paresis: one female: 11 years/2 month, GMFCS II and one male: 12 years/6 month, GMFCS III. Eight of the nine children had at least one previous treatment with Botulinum Toxin A in ST and semimembranosus muscle in the past (Table 1).

**Table 1:** Anthropometric and subject data ± standard deviation (range) and number of previous treatments with Botulinum toxin A.

Group	SP (n = 9)	TD (n = 9)	p
Age (year/month)	14/1±2/8 (10/7-18/2)	14/1±3/2 (10/0-18/5)	0.977
Gender (female/male)	5/4	5/4	
Body height (cm)	150.0±11.4 (136–176)	162.6±11.8 (147–182)	**0.036**
Body mass (kg)	43.1±11.1 (27.0–61.0)	51.2±9.9 (37.8–66.0)	0.122
Femur length (cm)	34.2±3.4 (31.3–41.0)	37.1±2.8 (33.3–40.8)	0.064
BMI	18.9±3.0 (13.9–23.2)	19.2±1.9 (17.1–22.3)	0.772
GMFCS (I-III)	II (3), III (6)	n/a	
Popliteal angle(degree)	71±6 (60–80)	n/a	
Maximal knee extension (passive) (degree)	27±10 (15–45)	n/a	
Number of previous treatment Botulinum toxin A (all longer than 6 month ago):		n/a	
M. Semitendinosus	0x(1), 1x(4), 2x(1), 3x(1), 6x(1), 8x(1)	n/a	
M. Semimembranosus	0x(1), 1x(4), 2x(1), 3x(1), 6x(1), 8x(1)	n/a	
M. Biceps femoris	0x(8), 1x (1)	n/a	
M Gracilis	0x(1), 1x(4), 3x(2), 6x(1), 8x(1)	n/a	
M. Psoas	0x(5), 1x (2), 4 (1), 6x(1)	n/a	
M. Rectus femoris	0x (6), 2x(2), 3x(1)	n/a	
M. Gastrocnemius	0x(4), 1x(2), 3x(1), 4x(1), 8x(1)	n/a	

TD = typically developing children, SP = spastic paresis; BMI = Body mass index, GMFCS = Gross Motor Function Classification System

### Measurements

#### Clinical measurements

Body mass and body height were measured and body mass index (BMI) was calculated (kg/m^2^). The popliteal angle was measured according to Reimers [35]. The passive maximal knee extension was measured using the neutral zero method [36]. For all knee angle measurements full knee extension is defined as 0° with an increasing angle towards knee flexion.

#### Measurements for knee moment angle characteristics at rest

**Fig 1:**
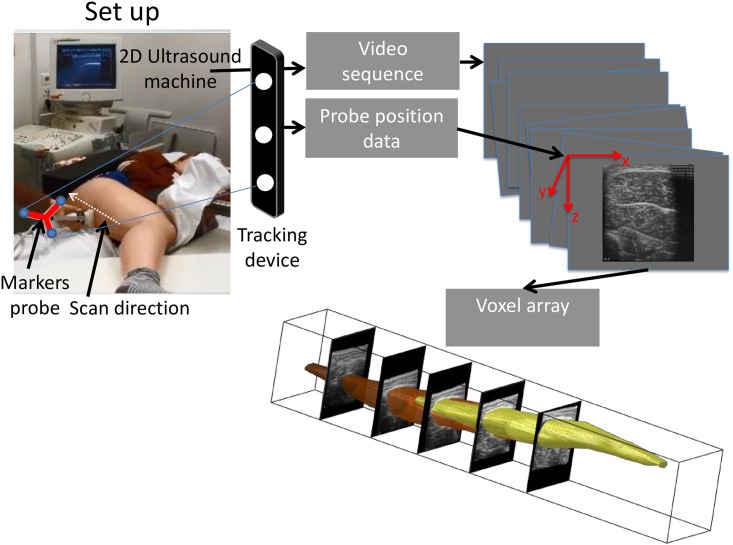
Setup of freehand three-dimensional ultrasound to measure semitendinosus (ST) muscle morphology. Subjects were positioned on an examination bed on their left side, with the hip of the measured (right) leg at 70° flexion. At knee angles corresponding to a knee moment of 0 and 4 Nm and at a knee angle of 65°, a 30–40 seconds video sequence of transverse US images was collected by a conventional 2D ultrasound apparatus, starting distally at the ST tendon to the ischial tuberosity (white arrow on the thigh indicates scan direction). The position of each ultrasound image in space was recorded by tracking the ultrasound probe (based on three markers that were rigidly attached to it—indicated by markers probe) using a motion capture system (tracking device). The images from the ultrasound video sequence were combined with the probe position data an reconstructed to a voxel array that was used for further analysis.

The experimental protocol and setup have been described in detail previously [31]. Subjects were positioned on an examination bed on their left side, with the hip of the measured (right) leg at 70° flexion (Fig 1). To prevent pelvic tilt and hip movements during measurements, pelvis and upper leg were tightly secured to the bed. The subjects stayed in this position for the entire measurement session. The lower leg was moved on a low-friction moveable plate. Changes in knee angle were measured using a Twin Axis digital goniometer (model SG150; Biometrics Ltd, UK). Anatomical bony landmarks (trochanter major, lateral femoral epicondyle, caput fibulae, and lateral malleolus) measured by an optically six marker rigid body Optotrak-probe (VUmc: Optotrak type 3020; Northern Digital, Waterloo, Canada) or 14 mm diameter-reflective VICON markers (UKBB: VICON MX20 System Oxford Metrics, Oxford, UK) defined the lengths of femur and fibula as well as the absolute knee angles. The latter were used for calibration of the goniometer, this was done to enhance the validity of the goniometer. Force applied at the lower leg was measured using a custom-made hand-held device instrumented with a bi-directional force transducer with an accuracy of 0.5 N (HBM Darmstadt, Germany). The lever arm was measured on the line between lateral femoral epicondyle and lateral malleolus, as the distance from the point of application of the force measurement device to the lateral femoral epicondyle. The lateral femoral epicondyle was used as an estimate of the location of the knee joint flexion/extension axis.

Activity levels of biceps femoris, gastrocnemius medialis, rectus femoris, and vastus lateralis muscles were assessed using surface electromyograpy (EMG). The skin was prepared and EMG electrodes placed according to SENIAM guidelines [37]. At the VUmc, force, knee angle, and EMG activity were sampled at 1000 Hz by a Mobi system (TMSI, The Netherlands). At the UKBB, force and knee angle were sampled at 100 Hz by a GSV-3USBx2-amplifier (ME-measuring systems GmbH, Germany) and EMG at 1000 Hz by a Biovision-EMG-system (Wehrheim, Germany).

The child watched a movie during the measurements for distraction and relaxation. Prior to the assessment of net knee moment-angle characteristics, the subject was asked to fully relax the leg for ten seconds to assess EMG activity at rest. To get accustomed to the measurements, three knee flexion-extension cycles from 110° of flexion to knee extension of maximal 20° were performed. The leg was moved only within a knee angle range that could be imposed without clearly detectable EMG bursts, as determined by visual inspection, and/or without discomfort experienced by the child. After these cycles, the lower leg was placed into a 110° knee flexion position and then slowly released till the plate stopped (i.e. zero knee moment). From that position, the knee was extended in steps of 5°. The subject was instructed to relax and neither to resist nor assist the movement. At each step of knee extension, the position was maintained for ten seconds to allow for effects of stress-relaxation. When the maximal knee extension angle was reached, the leg was slowly released and pulled towards flexion again. This procedure was repeated three times.

#### Measurements of ST morphology using 3D US

3D US imaging was performed at three knee angles (i.e. the angles corresponding to a knee flexion moment of 0 and 4 Nm, as well as at a knee angle of 65°). The angles at 0 and 4 Nm were selected during the three flexion-extension repetitions of the knee moment-angle measurements by determining the knee angle during every repetition and taking the mean of these three angles.

Then the subject’s lower leg was fixed by three suction cups around the low-friction moveable plate at knee angle corresponding to 0 Nm, while the bandage that stabilized the upper leg was released to allow for US scanning of ST. The order of US scanning at different knee angles was (1) angle corresponding to 0 Nm, (2) 65°, and (3) angle corresponding to 4 Nm. Two US scans were performed at each of the three knee angles. If during US scanning an EMG burst was detected and/or movement of the child was observed, the scan was discarded and an additional scan was obtained.

US imaging of ST was performed freehand using a B-mode US apparatus with a 5 cm linear probe 12.5 MHz (VUmc: Technos MPX, ESAOTE S.p.A., Italy; UKBB: Philips HD II). A 30–40 seconds sequence of transverse US images (i.e. axial plane of the ST) was collected starting distally at the ST tendon (i.e. at the point that the tendon could be sufficiently visualized in the popliteal fossa) to the origin on the ischial tuberosity (Fig 1). The US images were sampled at a rate of 25 Hz using an AD-video converter (Canopus ADVC-330, Grass valley) connected via a FireWire cable to the PC. The position of each US image in 3D space was recorded by tracking the US probe (based on three markers that were rigidly attached to it) using a motion capture system (VUmc: Optotrak type 3020; Northern Digital, Waterloo, Canada; UKBB: VICON MX20 System Oxford Metrics, Oxford, UK) (Fig 1). Positions of four bony anatomical landmarks (i.e. most prominent part of the ischial tuberosity, lateral and medial femoral epicondyles, and insertion of the ST tendon at the tibia) were recorded prior to scanning using an optically six marker rigid body Optotrak-probe (VUmc) or 14 mm diameter-reflective VICON markers (UKBB). All four bony landmarks were identified by palpation.

Prior to 3D US imaging the transformation matrix from the probe frame to the US images was determined by identifying a cross-point of two intersecting wires at a known position in a water cube within the US images (i.e. using the settings in scaling and resolution of the used ultrasound apparatus). The cross of the wire was scanned from different positions and with different tilt angles of the ultrasound probe, while the cross-wire was kept visible within the recorded ultrasound image sequence [38]. A custom made program in Matlab (version R2014A, the Mathworks Inc.) adapted from a previous version [39, 40] was used to fill a 3D voxel array with the US pixels (Fig 1). This algorithm consists of a distribution step and an additional gap filling step [41]. The size of a voxel was 0.2x0.2x0.2 mm.

### Data analysis

#### EMG data analysis

Since artefacts were present below 100 HZ, EMG data were high-pass filtered at 100 Hz [42, 43], rectified, and low-pass filtered at 5 Hz. Means and standard deviations (SD) of smoothed, rectified resting EMG were calculated. The threshold level for muscle activity was set at mean resting EMG + 2 SD.

#### Analysis knee-moment angle data

The analyses and reliability have been described in detail previously, reporting an standard error of the mean for these measurements of about 5° [31]. In brief, joint angle and force data were low-pass filtered at 1 Hz. For each knee angle (i.e. in steps of 5°) force and joint angle data were time averaged over the last three seconds of every ten seconds measurement interval. These last three seconds were taken for analysis because at these instance stress-relaxation of the muscles has attenuated and a steady state is reached. The net knee moment was calculated by multiplying the force measured at the force transducer by the lever arm.

Data were excluded from further analysis when the mean EMG envelope during the last three seconds of the ten second measurement interval exceeded the 2 SD threshold for one of the four muscles. The included data points from the three repetitions were fitted by a third order polynomial function. This equation was retrieved based on a stepwise regression analysis in a previous study [31]:

$$y=ax^{3}+bx^{2}+cx+d$$ (1)

In this equation, y represents the net knee moment, x represents the knee angle, and constants a, b, c, and d were determined by the fitting procedure. The minimal requirements to fit a function were defined as at least four data points (i.e. after the exclusion of data points due to the above described EMG threshold), at least one data point lower than 0.5 Nm and at least one data point higher than 3 Nm. Knee angles at 0, 0.5, 1, 2, 3 and 4 Nm (θ_0Nm_, θ_0.5Nm_, θ_1Nm_, θ_2Nm_, θ_3Nm_, θ_4Nm_) were derived from the fitted curves. Range of knee motion between 0 and 4 Nm was calculated (ROM_0-4Nm_). θ_0Nm_, θ_0.5Nm_, θ_1Nm_, θ_2Nm_, θ_3Nm_, θ_4Nm_, ROM_0-4Nm_ as well as maximum measured moment (*M*_max_) and maximum measured angle (θ_max_) were used for statistical analysis.

#### Image analysis 3D US

For US scanning the recorded EMG activity was checked to verify whether it did not exceed the 2 SD threshold. The following morphological characteristics of ST were determined (see for details of the method and validation [30]): length of the muscle-tendon unit (ℓmtu), muscle belly length (ℓm), distal tendon length (ℓt_dist_), total fascicle length (ℓfasc), fascicle length of the most proximal fascicle of the distal compartment (ℓfasc_dist_p_), fascicle length of the most distal fascicle of the distal compartment (ℓfasc_dist_d_), fascicle length of the most proximal fascicle of the proximal compartment (ℓfasc_prox_p_), whole muscle volume (*Vol*), muscle volume of the distal compartment (*Vol*_dist_), muscle volume of the proximal compartment (*Vol*_prox_) and, *PCSA*, defined as the cross-sectional area of all muscle fibers arranged in parallel including the intramuscular connective tissues.

Segmentation of ST for assessment of muscle volume was performed using Fiji (http://fiji.sc) [44, 45]. The outline of ST was encircled in transverse images every 5 mm along the length of the muscle [46]. The gaps between the encircled images were interpolated to obtain a fully segmented volume by Segmentation Editor Plugin (http://fiji.sc/Segmentation_Editor). Muscle volume of each compartment was measured using the volume measurement tool in Chimera 1.9 (http://www.cgl.ucsf.edu/chimera) [47]. *Vol*_dist_ and *Vol*_prox_ were summed to calculate *Vol*. Volume measurements were performed using the voxel array obtained at the 4 Nm knee angle. The coordinates in x, y, z directions of the following points were determined within the voxel array using Chimera 1.9: (1) most proximal and (2) distal ends of the tendinous inscription, (3) proximal end of the distal aponeurosis, (4) the distal end of the most distal fascicle (i.e. distal muscle-tendinous junction) and (5) ischial tuberosity. Determination of the ischial tuberosity was not always possible within the voxel array. When the ischial tuberosity was not visible within the voxel array, the position from the bony landmark registration was used for further calculations. Linear distances between coordinates of above mentioned points were used to define ℓm (point: 4–5), ℓfasc_dist_p_ (1–3), ℓfasc_dist_d_ (2–4), and ℓfasc_prox_p_ (1–5).

For estimation of distal tendon length the following procedure was performed. First, the most distal point of the distal tendon (proximal to the knee joint axis) was assessed within the voxel array. Subsequently, the following direction vectors were defined: (a) a line between the distal end of the most distal fascicle and the most distal visible point of the distal tendon (i.e. ‘line of tendon’) and (b) a line between the medial and lateral femoral epicondyles (i.e. ‘line of estimated knee joint axis’). The point along the ‘line of tendon’ from which the distance to the ‘line of estimated knee joint axis’ was smallest, was taken as estimate of the crossing of the distal tendon with the knee joint axis (6) [30]. The distance between the distal end of the most distal fascicle and the crossing of the distal tendon with the knee joint axis was calculated to estimate the distal tendon proximally of the knee axis (4–6). With the calculated crossing point and the registered coordinates of the insertion of the distal tendon on the tibia (7), the length of the distal tendon distally to the knee axis was assessed (6–7). The two tendon segments (i.e. proximally and distally of the knee axis) were summed to ℓt_dist_.

Voxel arrays were anonymized for group as well as for measurement condition (i.e. 0 Nm, 4 Nm, 65°) and were analyzed in randomized order. Assessment of the x, y, z coordinates of the seven above defined points was performed three times by the same observer (HH). Distances between the three identified points were calculated, yielding nine length measures per variable per subject. If the coefficient of variation (CV) of these nine values exceeded 10% of the mean of the length variable, the selected point related to this variable with the highest deviation with respect to the mean position was checked to verify whether the x, y, z, position of this point was misinterpreted. If this was the case, a new assessment for this specific point was made. When after these procedures the CV of the length measure still exceeded 10%, this length measure was excluded. For further calculations and statistical analyses, mean values of nine length measures were used.

To assess total fascicle length of ST (ℓfasc), ℓfasc_dist_p_ and ℓfasc_dist_d_ were summed. Length of the muscle-tendon unit (ℓmtu) was calculated by the sum of ℓm and ℓt_dist_. *PCSA* of ST was calculated by dividing *Vol* by ℓfasc at 4 Nm. Differences in length of total fascicle and tendon length between the 0 and 4 Nm condition were calculated (i.e. Δℓ_fasc_, and Δℓt_dist_) and expressed relative to the length measured at 0 Nm (i.e. Δℓ_fasc_^rel^ and Δℓt_dist_^rel^).

All length variables and differences herein were expressed as percentages of femur lengths (*variablename_*_norm_). Volume measures and *PCSA* were expressed as absolute values.

#### Statistics

Independent t-tests were used to test for differences between SP and TD children in anthropometric parameters, *M*_max_, θ_max_, ROM_0-4Nm_, morphological characteristics at 65° knee angle, muscle volumes, *PCSA*, and differences in total fascicle and tendon length. Within each group, paired t-tests were performed to test for differences in proximal and distal volume.

Differences in knee moment-angle characteristics and morphological characteristics (at 0 and 4 Nm) between SP and TD were tested with repeated measures ANOVA (factors: group x moment). Knee angles were tested at six knee flexion moments (θ_0Nm_, θ_0.5Nm_, θ_1Nm_, θ_2Nm_, θ_3Nm_, θ_4Nm_), while morphological characteristics were assessed at two flexion moments (knee angles corresponding to 0 and 4 Nm). For post hoc comparisons, independent t-tests with Bonferroni-Holm corrections were performed, accordingly the p-values were corrected. For all variables normality was tested by the Shapiro-Wilk test. If data was not normally distributed (which was the case for ℓt_dist_^65deg^_norm_ and ℓmtu^4Nm^_norm_) the non-parametric Mann-Whitney-U Test was used. For repeated measures ANOVA, the Greenhouse Geisser correction was used when the assumption of sphericity was violated. If homogeneity of variance was violated, Welch’s t-tests were used instead of independent t-tests.

To assess whether muscle morphological parameters can predict the mechanical properties at the joint level, multiple regression was used, including the data points of all subjects, to test the relationship between three predictor variables (i.e. *PCSA*, ℓt_dist_^0Nm^_norm_, and ℓfasc^0Nm^_norm_) and θ_4Nm_. In addition, for the combined group, correlations between ℓfasc^4Nm^_norm_ and θ_4Nm_ were calculated by Pearson correlation coefficient (Pearson’s r).

Data were presented as means ± SD and differences were presented as means ± standard error of difference. The level of significance was set at 0.05 for all statistical tests.

## Results

SP and TD children did not differ in body mass, BMI or femur length (Table 1). Body height was lower in children with SP (150.0±11.4 cm) compared to TD children (162.6±11.8 cm) (p = 0.036; Table 1).

### Knee moment-angle characteristics

**Fig 2:**
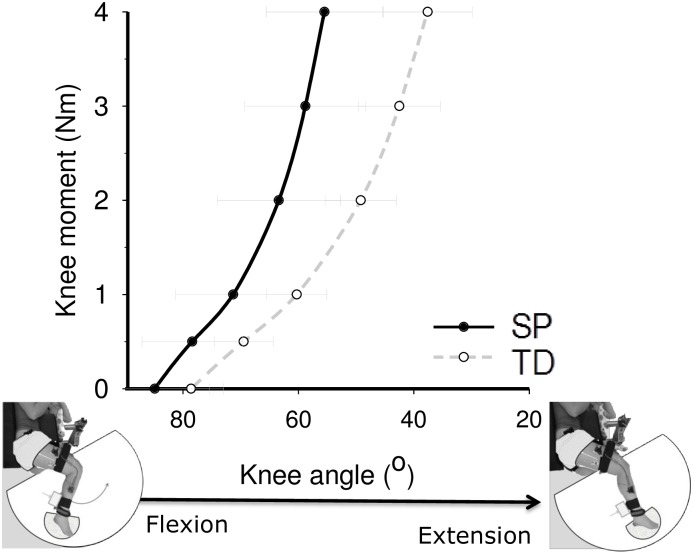
Knee moment-angle characteristics of children with a spastic paresis (SP) and typically developing (TD) children. The curve of SP children was shifted towards more flexed knee angles compared to the curve of TD children and has a steeper slope (i.e. higher stiffness). Black line: SP children; Grey dashed line: TD children. Values are mean ± SD.

Fig 2 shows for both SP and TD children net knee flexion moments as a function of knee angle. Repeated measures ANOVA for knee angle revealed an effect of group (p = 0.004) and an interaction effect of group and net knee flexion moment (p = 0.010, Fig 2). In TD children, knee angles measured at 0 Nm and 4 Nm were 78.6±5.6° and 37.6±7.7°, respectively. In the imposed posture (i.e. hip in 70° flexion), the maximal extended knee angle was 31.5±6.8°. For SP children, knee angles for the different moments were more flexed (θ_0Nm_: 84.9±9.5°, θ_4Nm_: 55.5±10.1°, and θ_max_: 54.1±11.8°). Maximum knee moment (*M*_max_) did not differ between groups (SP: 5.2±1.9 Nm; TD: 5.8±1.3 Nm, p = 0.446). Post-hoc t-tests revealed that in SP children knee angles corresponding to 0.5 Nm and higher were significantly more flexed than those of TD children (θ_0Nm_: p = 0.104; θ_0.5Nm_: p = 0.036; θ_1Nm_: p = 0.030; θ_2Nm_: p = 0.012; θ_3Nm_: p = 0.005; θ_4Nm_: p = 0.006; θ_max_: p<0.001). Differences in knee angles between SP and TD children increased with larger knee moments ranging from 8.9±3.4° for θ_0.5Nm_ to 17.9±4.2° for θ_4Nm_ and 22.7±4.5° for θ_max_. These results show that the knee moment-angle curve in children with SP was shifted towards more flexed knee angles in comparison to that in TD children. The significant interaction indicates also a steeper slope of the curve. This was also confirmed by a smaller ROM_0-4Nm_ in children with SP (29.4±5.4°) compared to TD children (41.0±8.1°) (p = 0.003) and, hence, a higher increase of knee angle per Nm in SP (SP: 0.14±0.03 Nm/°; TD: 0.10±0.02 Nm/°; p = 0.007).

### Morphological characteristics

The 3D US measurements for the 0 Nm condition were performed at a knee angle of 82.6±14.4° (SP) and 75.5±4.5 (TD) (p = 0.203) and for the 4 Nm condition at a knee angle of 55.6±11.9° (SP) and 38.3±7.5 (TD) (p = 0.004). The actual knee angle of 65° angle did not differ between the groups (64.3±6.5° for SP and 68.5±3.5° for TD, p = 0.127).

To characterize ST morphology, ℓmtu, ℓm, ℓt_dist_, *Vol*_prox_, and *Vol*_dist_ were analyzed in eight of the nine included children with SP and their matched TDs. In one child with SP, ultrasound measurements could not be performed due to anxiety and unrest. For this SP child, 3D US results of the matched TD child were also excluded from further analysis. In addition, fascicle length was excluded from analysis in a few children with SP due to inaccurate identification of x, y, and z coordinates of the required points (see method & Table 2).

**Table 2:** Morphological characteristics of semitendinosus muscle (ST) in children with a spastic paresis (SP) and typically developing (TD) children at 65^○^ knee angle and knee angles corresponding to 0 Nm and 4 Nm net knee flexion moments. P-value shows the difference between children with SP and TD children.

Morphological characteristics	n	SP	n	TD	p
ℓMTU^65deg^_norm_	8	121.2±5.7%	8	123.1±6.9%	0.553
ℓm^65deg^_norm_	8	74.6±6.6%	8	81.6±8.3%	0.084
ℓt_dist_^65deg^_norm_	8	46.6±7.1%	8	41.5±5.9%	0.161
ℓfasc^65deg^_norm_	7	38.2±5.1%	8	48.9±4.4%	**0.001**
ℓfasc_dist_p_^65deg^_norm_	5	19.9±3.7%	8	28.5±3.6%	**0.002**
ℓfasc_dist_d_^65deg^_norm_	7	26.8±6.0%	8	35.1±4.6%	**0.010**
ℓfasc_prox_p_^65deg^_norm_	7	19.5±3.4%	8	20.4±2.3%	0.586
ℓMTU^0Nm^_norm_	8	118.4±8.3%	8	121.7±5.2%	0.574
ℓMTU^4Nm^_norm_	8	126.7±8.7%	8	130.1±7.6%	
ℓm^0Nm^_norm_	8	72.1±7.9%	8	78.9±7.8%	0.055
ℓm^4Nm^_norm_	8	77.6±7.1%	8	86.8±8.5%	
ℓt_dist_^0Nm^_norm_	8	46.3±9.7%	8	41.8±5.1%	0.177
ℓt_dist_^4Nm^_norm_	8	49.1±7.8%	8	43.2±7.3%	
ℓfasc^0Nm^_norm_	6	37.2±3.8%	8	45.2±4.9%	**0.007**
ℓfasc^4Nm^_norm_	6	42.6±5.8%	8	53.5±6.2%	
ℓfasc_dist_p_^0Nm^_norm_	4	19.5±6.9%	8	26.0±2.8%	**0.025**
ℓfasc_dist_p_^4Nm^_norm_	4	21.0±5.6%	8	30.8±3.9%	
ℓfasc_dist_d_^0Nm^_norm_	8	28.5±8.3%	8	34.5±3.1	**0.024**
ℓfasc_dist_d_^4Nm^_norm_	8	28.2±6.8%	8	37.1±5.4%	
ℓfasc_prox_p_^0Nm^_norm_	7	17.8±4.4%	8	19.2±3.7%	0.543
ℓfasc_prox_p_^4Nm^_norm_	7	21.7±3.7%	8	22.7±4.1%	

ℓMTU = length of muscle-tendon unit; ℓm: length muscle belly; ℓt_dist_ = length of distal tendon ℓfasc = fascicle length; all length variables were expressed as % of femur length (_norm)_.

Comparison of origin and insertion distance (ℓmtu) of the ST at a knee angle of 65° should reveal the same values for both groups unless joint and/or bone morphology differ between groups. We found no difference in ℓmtu^*65deg*^_norm_ between SP and TD (Table 2). In SP children, both normalized fascicle lengths of the distal compartment (ℓfasc_dist_p_^*65deg*^_norm_, ℓfasc_dist_d_^*65deg*^_norm_) and normalized total fascicle length (ℓfasc^*65deg*^_norm_) assessed at 65° were 30.2±7.3%, 23.4.2±7.8%, and 21.8±5.0%, respectively lower than those in TD children. All other normalized length variables (ℓm^*65deg*^_norm_, ℓt_dist_^*65deg*^_norm_ and ℓfasc_prox_p_^*65deg*^_norm_) did not differ between SP and TD children (Table 2).

No differences between groups were shown for ℓMTU_norm_, ℓm_norm_, and ℓt_dist_norm_ when assessed at similar net knee moments (i.e. 0 and 4 Nm) (Table 2). Total fascicle lengths (ℓfasc_norm_) at 0 Nm and 4 Nm were 17.8±5.0% and 18.1±6.1% lower in SP than in TD children. This difference in ℓfasc_norm_ was mainly due to shorter fascicles in the distal ST compartment of SP children (ℓfasc_dist_p_norm_). The proximal fascicle length (ℓfasc_prox_p_norm_) did not differ between groups (Table 2).

**Fig 3:**
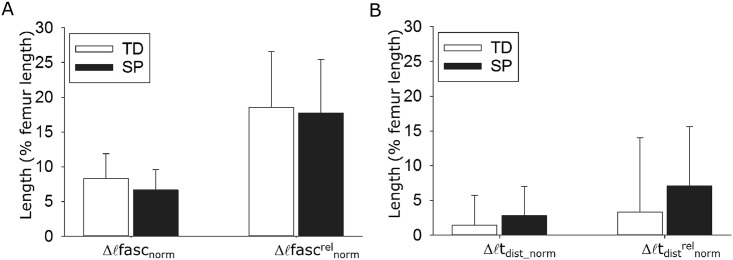
A: Absolute and relative (rel) length changes (Δ) of the fascicles between knee angles corresponding to 0 Nm and 4 Nm net knee moment. B: Absolute and relative length changes of the distal tendon between these two knee angles. Fascicle length and tendon length are normalized to femur length (ℓ_fasc_norm_, ℓt_dist_norm_). Absolute as well as relative length changes of fascicles and tendons did not differ significantly between children with a spastic paresis (SP) and typically developing (TD) children. Data are presented as means ± SD.

For the length variables, no significant interaction effects were shown between factors group and knee moment. This suggests that when the knee was extended from a knee angle corresponding to 0 Nm to a knee angle corresponding to 4 Nm, length variables in SP and TD children increased similarly. Absolute and relative fascicle and tendon length changes between 0 and 4 Nm did not differ significantly between groups (Δℓ_fasc_norm_: p = 0.372; Δℓ_fasc_norm_^rel^: p = 0.851; Δℓt_dist_norm_: p = 0.518; Δℓt_dist_norm_^rel^: p = 0.450; Fig 3).

**Fig 4:**
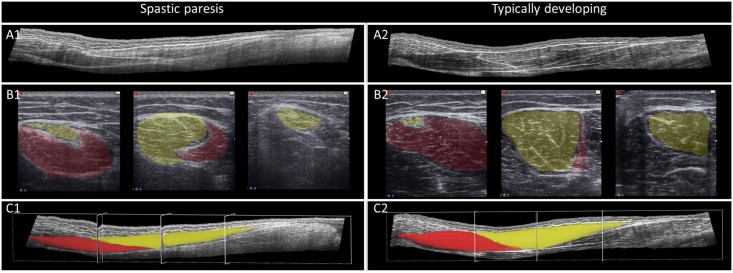
Typical example of 3D ultrasound images and segmentation of muscle volume of a child with a spastic paresis (left A1-C1) and typically developing child (right A2-C2). A: longitudinal view of semitendinosus muscle (ST) (proximal on the left side); B: transversal view of ST at three locations (most proximal on left side; orientation of images: medial (left), lateral (right)); yellow: distal compartment of ST; red: proximal compartment of ST; C: Proximal (red) and distal (yellow) compartments after segmentation.

**Fig 5:**
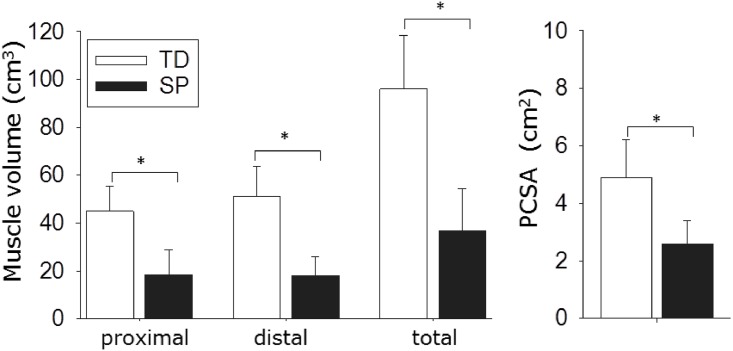
Muscle volume and physiological cross sectional area (PCSA) of semitendinosus muscle (ST) of children with a spastic paresis (SP) and typically developing children (TD). Muscle volume of ST (proximal, distal and total muscle volume) and PCSA are substantially smaller in SP children. PSCA was calculated by dividing muscle volume by fascicle length at 4 Nm. Data are presented as means ± SD; *p<0.01.

A typical example of a longitudinal view is shown in Fig 4. Total ST volume (*Vol*) was 62.0±10.5% smaller in children with SP (36.6±17.8 cm^3^) than in TD children (96.0±22.3 cm^3^) (p<0.001). This was also the case for each of the volumes of the proximal compartment (SP: 18.5±10.2 cm^3^, TD: 45.0±10.5 cm^3^; p<0.001) and of the distal compartment (SP: 18.1±8.0 cm^3^, TD: 51.0±12.6 cm^3^; p<0.001). Paired t-tests revealed that in TD children volumes of proximal and distal compartments (*Vol*_prox_ and *Vol*_dist_) differed (volume of distal compartment was 11.7±4.4% larger, p = 0.032). However, such a proximal-distal difference was not shown for the SP group (p = 0.802). PSCA was 47.7±12.1% smaller in SP compared to that in the TD children (p = 0.003, Fig 5).

### Relationship between muscle morphology and θ_0Nm_ and θ_4Nm_

**Fig 6:**
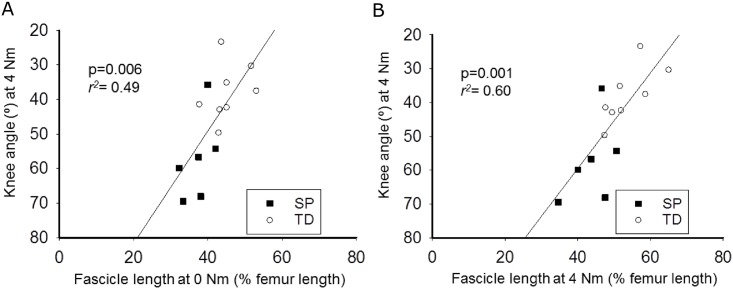
Knee angle at 4 Nm (θ_4Nm_) plotted as a function of normalized fascicle length at 0 Nm (ℓfasc^0Nm^) (A) and at 4 Nm (ℓfasc^4Nm^) (B). Variation in ℓfasc^0Nm^ and ℓfasc^4Nm^ explained a substantial part of variation in θ_4Nm_ (49% and 60%, respectively). Lines indicate the regression lines for the combined group. Separate symbols are used to indicate data points for SP (spastic paresis) and TD (typically developing).

Multiple regression analyses revealed that *PCSA*, ℓt_dist_^0Nm^_norm_, and ℓfasc^0Nm^_norm_ predicted θ_4Nm_ (*R*^*2*^ = 0.57, p = 0.033; θ_4Nm_ = 85.49-(1.30x*PCSA*)+(0.46xℓt_dist_^0Nm^_norm_)-(1.31xℓfasc^0Nm^_norm_)). However, only ℓfasc^0Nm^_norm_ significantly added to the prediction (p = 0.037). When *PCSA* and ℓt_dist_^0Nm^_norm_ were removed as predictor variables, ℓfasc^0Nm^_norm_ predicted θ_4Nm_ *r*^*2*^ = 0.49, p = 0.006; θ_4Nm_ = 114.20-(1.63xℓfasc^0Nm^_norm_), (Fig 6A). These results indicate that 49% of the variation θ_4Nm_ was explained by ℓfasc^0Nm^_norm_. Changing the prediction variable from ℓfasc^0Nm^_norm_ into ℓfasc^4Nm^_norm_ resulted in an explained variation of 60% for θ_4Nm_ (*r*^*2*^ = 0.60, p = 0.001; θ_4Nm_ = 116.03-(1.41xℓfasc^4Nm^_norm_), Fig 6B). These results indicate that the slope of the knee moment-angle curve was largely determined by the fascicle length.

## Discussion

This is the first study describing differences in knee moment-angle characteristics and ST morphology between children with SP, who were indicated for medial hamstring lengthening surgery, and TD children. The net knee moment-angle curve of children with SP in rest was shifted towards more flexed knee angles and showed a steeper slope. Muscle volume, PCSA, and fascicle length normalized for femur length of ST were all lower in SP compared to those in TD children. No differences in normalized muscle belly length of ST could be shown in SP compared to TD children.

### Relationship between knee moment-angle characteristics and ST morphology

The observed steeper slope and shift of the knee-moment angle curve in SP indicates a higher stiffness and decreased slack length of ST and/or other knee flexor muscles (i.e. m. semimembranosus, m. biceps femoris and m. gracilis). A stiffer MTU can theoretically be the result of an increase in the number of sarcomeres in parallel and connective tissues content (i.e. higher PCSA), a decrease in the number of sarcomeres in series, and/or decreased tendon length [48, 49]. In the SP group, PCSA of ST was substantially lower compared to that in TD, which suggests that in SP children, trophy of ST muscle fibers (i.e. cross-sectional growth of fibers) was attenuated. The substantial lower PCSA and the lower proximal and distal muscle volumes in children with SP is supported by previous studies reporting a lower ST muscle volume [26, 27, 29]. Note that the volume reduction in the current study (about 60%) exceeds that reported in previous studies (with about 35% volume reduction) [27, 29]. A reduced PCSA would decrease, rather than increase muscle stiffness. The substantially lower PCSA may be explained by a smaller cross-sectional area of muscle fibers (fCSA) or a lower number of muscle fibers in SP [50, 51]. Using ST biopsies, fCSA was shown to be 32% lower in children with SP compared to TD children [52]. This would partly explain the 48% PCSA reduction we observed, but suggest an additional reduction of muscle fiber number. The length of the distal ST tendon did not differ between groups in the current study and, hence, cannot explain the steeper slope of the knee-moment angle curve. In addition, shorter ST fascicles may have contributed to the observed changes in moment-angle characteristics. Assuming that for both groups at θ_0Nm_ the ST fascicles were at slack length, shorter fascicles at θ_0Nm_ in children with SP indicate fewer sarcomeres in series. This implies that at a given knee angle sarcomeres in fascicles of SP children will be more strained than those in TD. Thereby, passive fascicle stiffness will be higher. This may explain that the slope of the moment-angle curve in SP was steeper than in TD children. Shorter ST fascicles and fewer sarcomeres in series in ST of SP children are in agreement with the results of a previous study, in which *in vivo* sarcomere length in children with SP was increased (by 16% longer) compared that predicted by a musculoskeletal model for TD children [52]. Note however, that in the latter study the patient group consisted of a higher number of non-ambulatory children [52]. Therefore, a higher fascicle stiffness may be more likely to be found in this group compared to the SP children in the current study, who were all ambulant.

As our results indicate that fascicle length cannot explain all variation in θ_4Nm_, also other mechanisms should have contributed to the steeper knee-moment knee angle curve in children with SP. An increased fiber stiffness due to changes in the composition of the sarcomeres (i.e. change in titin isoform expression) has not been found for ST in SP [52]. However, dissected muscle fascicles of ST in SP children were less compliant at sarcomere lengths above about 3.5 μm compared to TD [52], suggesting altered mechanical properties of fascicles in SP children. However, in the current study relative changes in fascicle length between 0 and 4 Nm in the SP and TD group were not different, indicating no differences in fascicle stiffness between the groups. With knee angles measured at 4 Nm knee flexion moment, sarcomeres may not have been stretched above 3.5 μm and as such it is not likely that altered mechanical properties of SP muscle fascicles explain our observed changes in the knee moment-ankle characteristics.

Alternatively, enhanced resistance to stretch may be caused by increased stiffness due to changes in intramuscular connective tissues. Differences in collagen content were reported from muscle biopsies of ST [52] and other muscles of children with SP [53, 54]. Accumulation of connective tissue was reported to occur mainly around the neurovascular tract [54]. A higher ultrasound echo intensity was reported within the medial gastrocnemius of SP children [55]. It was shown that such echo intensity was related to the amount of fibrous tissue (i.e. collagen) and fatty tissue [56, 57]. Collagen content, as mentioned above, as well as fat content were reported to be increased in children with SP [52–54, 58]. In this study we did not quantify echo intensity, but observed major differences between images of SP and TD children with more white pixels and less contrast in images of SP children (see for typical example Fig 4). This suggests differences in connective tissue composition of ST in our group of children with SP compared to that of TD children, which may enhanced MTU stiffness by increasing the intramuscular connective tissue arranged in parallel of the muscle fibers. Note however, that quantifying echo intensity does not allow to distinguish between connective and fatty tissue [56]. Therefore, muscle biopsies studies in combination with mechanical and morphological measurements *in vivo* are required to assess the impact of tissue composition on MTU stiffness.

Besides MTU stiffness of ST, or other knee flexor muscles, altered epimuscular myofascial interactions [59–61] as well as differences in other structures around the knee (e.g. articular capsule, ligaments, nerves, blood vessels, and connective tissues) may also contribute to the steeper slope of knee-moment angle curve in children with SP.

When measuring net knee flexion moments, this includes contributions from agonistic and antagonistic muscles, as well as above mentioned non-muscular structures around the joint [62]. Shorter fascicle lengths of rectus femoris muscle, measured at the resting angle of the knee, of SP children have been reported [63]. It is likely that, besides knee flexion muscles, also knee extension muscles in our group of SP children were affected. As the measured net knee moment is based on the net mechanical effects of agonist and antagonist muscles, shorter fascicles of a knee extensor muscle (i.e. rectus femoris muscle) would shift the knee angle at which a net zero knee moment (equilibrium) was attained to a more extended knee angle. This would imply that for these children at θ_0Nm_ ST was lengthened beyond its resting length and that at 0 Nm fascicles of ST were not at their passive slack length, indicating that the difference in actual resting fascicle length between SP and TD children was even more pronounced.

### Morphological differences between ST compartments

Our results suggest that there are local differences in the effects of SP on ST morphology. The effects were greater for the distal compartment than for the proximal compartment, particularly regarding the shorter fascicle length. Shorter fascicles within the distal compartment in children with SP may be secondary to the joint position in which ST is predominantly used during movement. Flexed knee gait is characterized by excessive hip and knee flexion during stance [11, 64, 65] and is often associated with a lack of knee extension in terminal swing [66]. Therefore, during terminal swing ST is unstrained due to a lack of knee extension [66]. In addition, strain applied to ST during gait may not be equally distributed between proximal and distal compartments of ST. This means that the hip flexion may stretch proximal fascicles of ST, while fascicles of the distal compartment may be at relatively shorter length due to knee flexion. However, this conclusion regarding differences in altered fascicle lengths between compartments due to a flexed gait pattern may only occur if effects of ST over hip and knee are to some extent independent [25]. This independency of compartments is plausible because it is known that the compartments have separate innervations [20–25] and there are possible enhanced [59–61] epimuscular myofascial linkages between adjacent muscles or extramuscular connective tissues [67, 68].

### Clinical implications

Given the relation between PCSA and force generation [69], the reduced PCSA observed in this study and the reduced muscle volume of ST (present study and [26, 27, 29]) suggest that, compared to TD children, in a majority of children with SP ST is weak. As lengthening of the ST tendon is presumed to induce even more ST weakness [16], a low preoperative PCSA of ST (i.e. more than 60% lower than in TD) may be a risk factor for side effects of the surgery such as increased anterior pelvic tilt during standing and walking. In addition, fascicles that are short before surgery may further decrease in length after surgery if due to lengthening of the tendon the muscle belly is not sufficiently strained. Consequently the active length range of force exertion of the muscle will decrease. Therefore, caution should be taken before indicating hamstring lengthening. Surgical lengthening of ST tendon in SP may result in a shift of the knee-moment angle curve towards more normal knee extension. In addition, the longer and more compliant tendon, may also result in a decrease in slope of the knee moment-angle characteristics. These effects may be beneficial for knee movement during gait, but may also increase hip flexion and anterior pelvic tilt, which has been reported to occur after SEMLS surgery [8, 10, 14, 15]. To what extent the morphological and mechanical alterations in children with SP prior medial hamstring lengthening contribute to the treatment outcome needs to be evaluated in follow-up studies.

### Limitations

The number of subjects in the current study was rather low and consisted of a selected group of children with SP (i.e. selected for surgery and all ambulant, GFMCS I-III). Therefore, results cannot be generalized to the whole population of children with SP. However, as our study group is suspected to be limited by hamstring muscles during gait (and thus indicated for surgery), the current results can be assumed to be representative for the pre-surgical situation of hamstring lengthening in children with SP.

The children with SP were included and assessed in two medical centers. The positioning of the subjects in the measurement set-up was identical in the two centers. However, measurements were performed with different ultrasound apparatus’, and motion tracking, force measurement and EMG systems. Although all systems are high-end and commonly used in clinical practice and for research, this could have resulted in additional variance. To reduce variation, the same person (HH) performed all but one measurements and analyzed all data.

Ultrasound measurements do not allow to measure sarcomere length and/or number of sarcomeres in series within muscle fibers. Although fascicle length measured at standardized knee flexion moments may indirectly provide information about sarcomere length and number, for accurate assessment of these parameters fascicle length as well as sarcomere length should be measured in the same child [70].

## Conclusions

Knee moment-angle characteristics and ST morphology in children with SP differ from those in TD children. Overall, our results indicate a strongly reduced ST cross-sectional area in children with SP (i.e. 48% smaller PCSA) which is likely due to a reduced rate of muscle trophy. The 18% shorter fascicle length in children with SP suggest a reduced number of sarcomeres in series. Shorter fascicles of ST explain a substantial part of the limited knee ROM in children with SP. Regarding their effects on ST stiffness, shortening of fascicles and reduction in PSCA are canceling each other and may not fully explain the increased slope of the net knee flexion moment–angle characteristics in children with SP.

## Supporting Information

S1 Table Data. Supplementary table containing all individual data from which the summary data are presented in the manuscript. Absolute data as well as normalized data are included in the supplementary table.
(XLSX)
